# A Case of Hysteria Complicated with Mania-a-Potu

**Published:** 1855-11

**Authors:** Thos. S. Denny

**Affiliations:** Atlanta, Georgia


ARTICLE II.—*A Case of Hysteria complicated with Mania-a-Potu.* By THOS. S. DENNY, M. D., Atlanta, Georgia.

A case of some interest having occurred to me recently, it is presented for the columns of your Journal:

The patient was a woman of about 35 years of age; had been married some years, but had never borne offspring. On the 26th of June last she had an attack of very violent pain in the abdomen. She stated that the catamenial discharge had appeared the previous week, but this did not prevent me from entertaining suspicions as to some irregularity of the menstrual functions. The pulse was rather slow, but full and regular throughout the attack, and scarcely at all affected by the recurrence of the most violent pains. The skin was cool and moist; no headache; the tongue was clean and the eyes natural, except under the circumstances hereafter mentioned.

She was put upon the use of a mixture of the Tinctures of Valerian, Opium, and Capsicum, with the Aqua, Menth. Piparita, and Spit. Ether Nit., and though the pain was somewhat alleviated, occasionally they were exceedingly violent, so much so as to compel me to administer large doses of Laudanum—40 to 50 drops—three or four times a day.

These large and repeated doses produced no increase of the pulse. On the fourth day, the bowels being constipated, ℥ ss. Ol. Ricini was administered, which produced one evacuation, the Laudanum being suspended. About this time symptoms manifested themselves which I looked upon as Mania-a-Potu, *knowing* that she was intemperate, for the relief of which the Laudanum was renewed, and with entire success, at the same time commanding the pain, which still recurred occasionally, though at longer intervals.

The appearance of the Mania was very sudden, and was so exceedingly violent as to make it difficult to control her at all.

On the fifth day the catamenia appeared, and immediately all violent symptoms vanished, and by the second of July the patient was as well as usual, and able to attend to all her duties, which were of a laborious character.

I regard the peculiarity of this case to consist in the very great variety of symptoms manifested in a short time, and exhibiting, as I think, a combination of Mania-a-Portu with Hysteria; for while many of the symptoms occurring in the case are of course attributable to a hysterical condition ot the system, I am satisfied that there were others pointing out as distinctly Mania-a-Portu, and which were in perfect accordance with the *known* previous habits of the patient.

